# Annual and Seasonal Changes in Parasitism Rates by *Hadronotus Pennsylvanicus* (Hymenoptera: Scelionidae) on the Squash Bug *Anasa Tristis* (Hemiptera: Coreidae) in Squash Fields: Implications for Augmentative Releases

**DOI:** 10.3390/insects13110984

**Published:** 2022-10-26

**Authors:** Mary L. Cornelius, Bryan T. Vinyard, Donald C. Weber

**Affiliations:** 1 Invasive Insect Biocontrol and Behavior Laboratory, USDA Agricultural Research Service, Beltsville Agriculture Research Center, 10300 Baltimore Ave, Building 007, Beltsville, MD 20705, USA; 2 Statistics Group, USDA Agricultural Research Service, Northeast Area Office, Beltsville, MD 20705, USA

**Keywords:** *Gryon pennsylvanicum*, biological control, egg parasitism

## Abstract

**Simple Summary:**

The squash bug is a serious insect pest of squash and other cucurbit crops in North America. Squash bugs damage the crop by feeding on plant leaves, stems, and fruits, leading to significant reductions in fruit yield with economic losses for growers. While use of insecticides helps lower squash bug numbers, it can have negative effects on the environment and on important beneficial insect species like pollinators and other beneficial insects. Alternatives such as biological control are not well studied for squash bugs. The squash bug has an important natural enemy, the native parasitoid wasp Hadronotus pennsylvanicus, which attacks the squash bugs’ eggs. From 2016 through 2021 in Maryland, we monitored parasitism of squash bug eggs through the season on summer squash crops. Although the overall parasitism rate was about 11%, this differed greatly by year and over the course of the season, suggesting that augmentation (rearing and releasing the beneficial wasps) in early summer could be valuable in most years, and that this effect would persist over the season. This natural enemy and its potential management for biological control of squash bugs may assist vegetable farmers, pest managers, and researchers involved with squash and related cucurbit crops.

**Abstract:**

This study evaluated parasitism rates by *Hadronotus pennsylvanicus* (Ashmead) (Hymenoptera: Scelionidae) on the squash bug *Anasa tristis* DeGeer (Hemiptera: Coreidae) over a six-year period in squash fields in Maryland. From 2016–2021, 2226 wild squash bug egg masses were collected, 2180 (98.0%) *A. tristis* egg masses and 46 (2.0%) *A. armigera* egg masses. The mean (±SE) parasitism rate was 10.9 ± 0.16%. Yearly parasitism rates were significantly different with rates in 2017 and 2018 that were significantly lower than in 2019, 2020, and 2021. The significant difference in parasitism rates based on planting date was primarily due to the high parasitism rate observed in 2021. These results suggest that the use of augmentative releases early in the season could result in effective control by increasing parasitism earlier in the season and by causing the parasitism rate in the field to peak at a higher number late in the season.

## 1. Introduction

The squash bug, *Anasa tristis* (De Geer) (Hemiptera: Coreidae), is a major pest of cucurbit crops [1]. Numerous studies have evaluated the effects of egg parasitism on field populations of *A. tristis* [2,3,4,5]. In the mid-Atlantic region, *Hadronotus pennsylvanicus* (Ashmead) (formerly known as *Gryon pennsylvanicum* (Ashmead)) [6] is the predominant egg parasitoid of *A. tristis*. In a two- year field study in Maryland, *H. pennsylvanicus* accounted for over 99% of egg parasitism and the average parasitism rate was 55.7% for wild eggs and 21.8% for sentinel eggs [3]. In Virginia, the average egg parasitism rate on wild egg masses was 66% [5]. *Anasa armigera* Say (Hemiptera: Coreidae) is also found in squash fields in Maryland and comprised 4.3% of the total squash bug egg masses collected in 2016–2017; parasitism rates on *A. tristis* and *A. armigera* were not significantly different [4].

Augmentative releases have been used to increase the efficacy of egg parasitoids as biological control agents [7,8]. The use of augmentative releases of *H. pennsylvanicus* to control squash bugs has been evaluated in experimental plots of pumpkin. Squash bug densities were significantly lower in plots where *H. pennsylvanicus* parasitoids had been released [2]. Boyle [9] augmented *H. pennsylvanicus* in the early season in southeastern Virginia, resulting in significantly increased parasitism in certified organic cucurbit plantings, compared to plantings where the parasitoid had not been released. The efficacy and impact of such augmentative releases as a biological control tactic may be affected by variations in the natural populations of parasitoids, both regionally and year-to-year.

Egg parasitism rates on *A. tristis* egg masses vary annually, seasonally, and geographically. In a two-year study conducted in Kentucky, egg parasitism by *H. pennsylvanicus* ranged from 0–31% in collections of *A. tristis* egg masses on different dates in 2005 and 2006 [10]. Parasitism rates on *A. tristis* egg masses collected weekly in 2013 and 2014 at the USDA ARS Beltsville Agricultural Research Center (BARC) in Maryland ranged from 0% of eggs parasitized in the first week of July to 72.8% of egg parasitized in the last week of July [3]. In both 2016 and 2017, egg parasitism rates at BARC increased over the season, peaking in the first week of August at 24.7% in 2016 and in the last week of August at 45% in 2017 [4]. In a survey of egg parasitism by *H. pennsylvanicus* on *A. tristis* egg mases collected from 30 counties in Virginia, parasitism ranged from 0–100% of egg masses with 39.4% of egg masses parasitized in 2014 and 49.4% parasitized in 2015 [5].

Planting date may influence egg parasitism rates. Field studies have found that *A. tristis* populations reach higher levels in fields with an earlier planting date than a later planting date [11,12]. In two previous studies, egg parasitism rates were calculated from wild egg masses collected in experimental plots in squash fields at BARC. In the first study, squash fields were planted in mid-May, parasitized eggs were first collected in the second week of July, and the egg parasitism rate increased rapidly, peaking in the last week of July with 72.8% of eggs parasitized [3]. In the second study, squash fields were planted in mid-June, parasitized eggs were also first collected in the second week of July. However, egg parasitism rates increased slowly, reaching an average of only 9.8% at the end of July and an average of 37.7% in the last week of August [4]. The results of these two studies conducted at BARC indicate that there may be significant differences in parasitism rates of *H. pennsylvanicus* in different years and that planting date could potentially be a factor influencing parasitism rates of squash bugs.

Parasitism rates can also be influenced by host density. Although parasitism was density-dependent in some field studies [13], it was density-independent or inversely dependent in others [14,15]. The host density of wild *A. tristis* egg masses appeared to influence parasitism rates on sentinel squash plants in squash and cucumber fields [16].

The objective of this study was to monitor annual and seasonal fluctuations in the parasitism rate of squash bug egg masses in squash fields in Maryland over a six-year period and to evaluate the effect of planting date on parasitism rates. Understanding annual and seasonal patterns in naturally occurring parasitism rates in squash fields is critical in order to improve strategies to use augmentative releases to increase the overall efficacy of *H. pennsylvanicus* as a biological control agent of squash bugs.

## 2. Materials and Methods

### 2.1. Experimental Site

Squash fields were located at the North Farm of BARC, Beltsville, MD (39°01′056″ N, 76°55′054″ W). The sizes of the squash fields ranged from 0.30–0.55 ha. The fields had been planted with rye (*Secale cereale* L.) as a winter cover crop; squash was seeded in no till fields with a 76 cm distance between rows. On 12–14 June 2016 and 28–30 June 2017, four squash fields were planted with *Cucurbita pepo* cv. ‘Slick Pik YS26’. In 2018–2021, squash fields planted with *C. pepo* cv ‘Yellow Crookneck’: in 2018, four fields on 16 June; in 2019, two fields on 20 May and two fields on 19 June; in 2020, four fields on 1 June, and in 2021, one field on 28 May and a second on 30 June. The late fields were planted adjacent to the early fields. In each field, egg masses were collected weekly from 5 m by 5 m plots with each plot containing approximately 100 squash plants. In 2016 and 2017, there were two plots per field, one on each end. In 2018–2021, there was one plot located in the center of each field.

### 2.2. Collection of Wild Squash Bug Egg Masses

All plants in the experimental plots were thoroughly searched weekly for wild egg masses of *A. tristis* and *A. armigera*. Egg masses were collected and brought back to the laboratory. Eggs were counted and any eggs damaged by chewing predators were recorded. Egg masses were placed in Petri dishes and kept in a growth chamber (25 °C and a photoperiod of 16:8 [L:D] h) to record nymphal hatch or parasitoid emergence. After 30 days, any intact eggs were dissected and those containing fully developed unemerged parasitoids were recorded.

### 2.3. Statistical Analysis

Egg parasitism rates were estimated for each level of the main-effects: planting date, year, and week; and for the nested effects of week-in-year and year-in-week. Because of the nested structure of the effects, three different specifications of the ANOVA model were necessary to obtain all comparisons of interest. The ANOVA model for the planting date effect contained the three fixed effects: planting date, year-in-planting date, and week-in-planting date-and-year. The other two ANOVA models each contained two fixed effects, respectively: year and week-in-year; and week and year-in-week. To satisfy ANOVA normality requirements, the square root then arcsine transformations were applied to the parasitism rate observed on each egg mass. Egg parasitism rates among egg masses observed at the six times in the same year exhibited a heterogeneous first-order auto-regressive covariance structure; included in each of the ANOVA models using SAS PROC MIXED with TYPE = ARH(1) specified in the REPEATED statement [17]. Pairwise means comparisons were obtained using LSMEANS statements, with the appropriate SLICE option for each nested effect, and with the option ADJUST = SIDAK to ensure experiment-wise α = 0.05 and using the PDMIX800 macro [18]. Estimates of means and lower and upper confidence limits were obtained from the ANOVA models and back-transformed to the original percentage parasitism rate scale. Because 66% of the normal distribution curve is between the mean ± 1 standard error, standard errors were estimated as the difference between the upper 66% confidence limit and the mean, on the percentage scale. Annual host density estimates were obtained from an ANOVA model with year and week-nested-in-year fixed effects, specifying a negative binomial distribution and log link, using SAS PROC GLIMMIX [17].

## 3. Results

From 2016–2021, 2226 wild squash bug egg masses were collected, 2180 (98.0%) *A. tristis* egg masses and 46 (2.0%) *A. armigera* egg masses. Out of a total of 37,709 eggs, 4123 eggs were parasitized. The mean (±SE) parasitism rate for 2016–2021 was 10.9 ± 0.16%. Of the 4123 eggs parasitized, *H. pennsylvanicus* accounted for 3777 (91.6%), and *Ooencyrtus anasae* (Ashmead) accounted for 346 eggs (8.4%) (complete data in Table S1).

The number of plots sampled varied over the years, ranging from 43 in 2016 to only 6 in 2020 and 2021. The total number of egg masses collected in each year ranged from 534 in 2016 to 29 in 2021 (Table 1).

**Figure 1:**
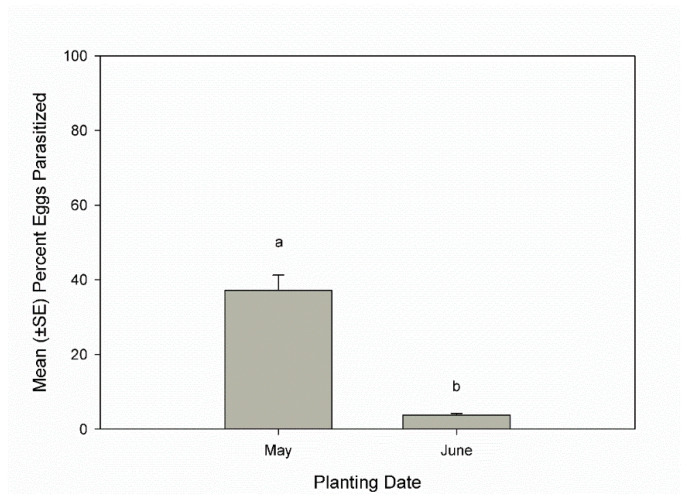
Mean (+SE) percent parasitism on egg masses in squash fields planted in May or June, 2016–2021, Beltsville, Maryland, USA. Weeks topped by the same letters were not significantly different (ANOVA: experiment-wise *p* ≤ 0.05).

**Figure 2:**
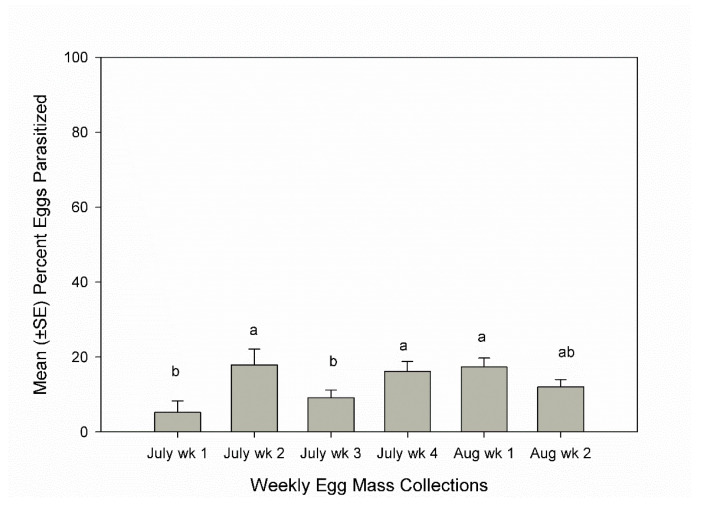
Mean (+SE) percent parasitism on egg masses collected weekly from squash fields, 2016–2021, Beltsville, Maryland, USA. Weeks topped by the same letters were not significantly different (ANOVA: experiment-wise *p* ≤ 0.05).

The host density of egg masses per plot (25 m²) varied per year (F _5, 90_ = 293, *p* < 0.0001), with the highest density in 2020 of 67.2 ± 17.6 and the lowest density in 2021 of 3.9 ± 1.3. Host density did not have a significant linear relationship with parasitism rates (F _1, 1694_ = 2.15, *p* = 0.14). Yearly parasitism rates were significantly different (F _5, 1023_ = 32.2, *p* > 0.0001) with rates in 2017 and 2018 that were significantly lower than in 2019, 2020, and 2021. The significant difference in parasitism rates based on planting date (F _1, 837_ = 116.5; *p* < 0.0001) was primarily due to the high parasitism rate observed in 2021 (Figure 1). Overall, parasitism rates increased over the season (F _5, 786_ = 5.1; *p* < 0.0001) and were significantly higher in the second and fourth weeks of July and the first week of August than in the first or third weeks of July (Figure 2).

**Figure 3:**
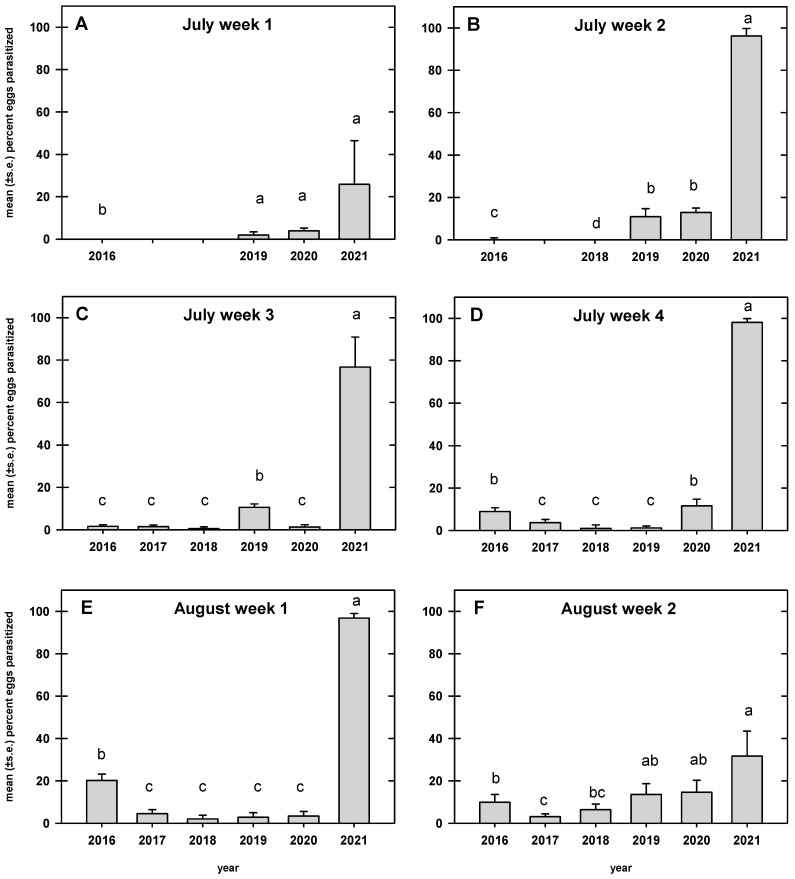
Mean (+SE) percent parasitism for each week compared by year, Beltsville, Maryland, USA. For each week, years followed by the same letters were not significantly different (ANOVA: experiment-wise adjusted *p* ≤ 0.05).

There was variability in the weekly parasitism rates compared by year (F _22, 1385_= 6.4; *p* < 0.0001). In every week, parasitism rates were significantly higher in 2021 than in 2016, 2017, and 2018. However, parasitism rates in 2019 and 2020 were not significantly different than in 2021 in either the first week of July or the second week of August (Figure 3).

## 4. Discussion

Augmentative releases of egg parasitoids have been used effectively to control pests in vegetable crops [8]. Boyle [9] released *H. pennsylvanicus* as parasitized *Anasa tristis* eggs in the early season in southeastern Virginia, resulting in significantly increased parasitism in certified organic cucurbit plantings, compared to control farms, in two separate years. Augmentative releases of *H. pennsylvanicus* are also being considered to control the invasive seed pest *Leptoglossus occidentalis* Heidemann in Europe [19]. Information on the annual and seasonal fluctuations in the natural populations of *H. pennsylvanicus* in squash fields will be useful for developing effective biological control programs using augmentative releases.

There were significant differences in parasitism rates between years. Parasitism rates were significantly affected by planting date, but the difference was primarily due to the high parasitism rate detected in 2021 where the sample size was small. Therefore, more research is needed to evaluate the effect of planting date on parasitism rates by *H. pennsylvanicus* on squash bug egg masses.

There was no significant effect of host density on parasitism rates. It is possible that parasitism rates are affected by the host density in the previous year. In a year with high host density, the number of parasitoids emerging from hosts and subsequently overwintering as adults would be expected to be higher, which would result in higher numbers of parasitoids emerging in the spring. In 2020, host density was very high, but parasitism was low. In 2021, parasitism was high starting in the first week of July, suggesting that the number of overwintered parasitoids migrating into squash fields may have been higher than in other years. However, the number of egg masses collected was very low. Further research is necessary to evaluate whether host density in the previous year influences the migration of overwintering parasitoids into squash fields.

The overwintering requirements and limitations of *H. pennsylvanicus* are not well known [20]. Scelionids that overwinter as adults, such as *H. pennsylvanicus* and many other species in this family, must survive several months without host eggs in temperate climates [21]. Annual cropping systems oftentimes do not provide adequate habitat for overwintering parasitoid adults [22]. Local variation in winter refugia has been shown to be important for other parasitoids overwintering as adults [23], as has the availability of food resources, e.g., [24]. Furthermore, synchrony of emergence of hosts and parasitoids can have a strong influence on scelionid mortality and fecundity, and resulting efficacy of biological control [21]. Better knowledge of these multiple factors affecting overwintering abundance and survival would help to explain the variability of early season abundance of *H. pennsylvanicus*, as well as to improve their effective conservation and augmentation.

The average parasitism rate at BARC from 2016–2021 was only 10.9%. Therefore, augmentative releases would be necessary in order to use *H. pennsylvanicus* as a biological control agent to achieve greater pest suppression. Overall, parasitism was higher in the second week of August than in July. In a previous study conducted at BARC, parasitism gradually increased from the first week in July, peaking in late July at 72.8% [3]. These results where the parasitism rate gradually increases over the season indicate that females emerging from host eggs in a squash field are likely to search for host eggs within the squash field rather than dispersing. Therefore, the use of augmentative releases early in the season could result in effective control by increasing parasitism earlier in the season as well as by causing the parasitism rate in the field to peak at a higher number later in the season.

## Figures and Tables

**Table 1:** Mean (±SE) percent squash bug eggs parasitized per year from 2016–2021.

Year	Number of Plots Sampled	Number of Egg Masses Collected	¹ Number of Egg Masses per Plot	² Mean (±SE) Percent Eggs Parasitized
2016	43	534	9.7 ± 1.2 D^3^	4.7 ± 1.1 BC
2017	32	449	14.7 ± 1.8 C	3.1 ± 0.7 CD
2018	20	300	14.6 ± 2.2 C	1.5 ± 0.6 D
2019	21	463	21.5 ± 3.4 B	5.9 ± 1.0 B
2020	6	451	67.2 ± 17.6 A	7.0 ± 1.1 B
2021	6	29	3.9 ± 1.3 E	76.6 ± 6.5 A

^1^ Estimates obtained from a Negative Binomial ANOVA model. ^2^ LSMeans estimates obtained from a Normal ANOVA model fit to Arcsine sqrt proportion parasitized. ^3^ Year means with the same letter were not statistically different using Sidak-adjusted pairwise means comparisons with *p* > 0.05.

## Data Availability

All data collected in this study are available in Supplementary Material (Table S1).

## Supplementary Materials

The following supporting information can be downloaded at: https://www.mdpi.com/article/10.3390/insects13110984/s1. Table S1: Row date.
